# An *rhs* Gene Linked to the Second Type VI Secretion Cluster Is a Feature of the Pseudomonas aeruginosa Strain PA14

**DOI:** 10.1128/JB.00863-13

**Published:** 2014-02

**Authors:** Cerith Jones, Abderrahman Hachani, Eleni Manoli, Alain Filloux

**Affiliations:** MRC Centre for Molecular Bacteriology and Infection (CMBI), Department of Life Sciences, Imperial College London, London, United Kingdom

## Abstract

The type VI secretion system (T6SS) of Gram-negative bacteria has been involved in various processes, notably bacterial competition and eukaryotic cell subversion. Most Pseudomonas aeruginosa strains possess three T6SS gene clusters, but only the function of the first T6SS (H1-T6SS) has been clearly elucidated. It is involved in the secretion of three toxins (Tse1 to -3) that target bacterial competitors. In the case of the H2- and H3-T6SS, no clear function has been assigned, and only one effector has been associated with these systems. Yet the H2-T6SS was proposed to promote P. aeruginosa internalization in nonphagocytic epithelial cells. Although the H2-T6SS genetic organization is conserved across P. aeruginosa isolates, one feature is the presence of an additional transcriptional unit in the PA14 strain H2-T6SS cluster, which is divergent from the core H2-T6SS genes. A specific set of four genes encodes an Hcp protein (Hcp2), a VgrG protein (VgrG14), an Rhs element (PA14_43100 or RhsP2), and a protein with no homologies with previously characterized proteins (PA14_43090). In this study, we engineered a P. aeruginosa PA14 strain carrying an arabinose-inducible H2-T6SS on the chromosome. We showed that arabinose induction readily promotes assembly of the H2-T6SS, as seen by monitoring Hcp2 secretion. We further studied the secretion fate of VgrG14 and RhsP2, but these were not detectable in the extracellular medium. We finally investigated whether activation of the PA14 H2-T6SS gene cluster could influence phenotypic traits such as internalization in eukaryotic cells, and we reported noteworthy differences compared to strain PAO1, which may be accounted for by the described genetic differences.

## INTRODUCTION

Pseudomonas aeruginosa is a Gram-negative bacterium that is an opportunistic pathogen equipped with a wide range of protein secretion systems (1). These systems are named by type, i.e., the type I (T1SS) to type VI (T6SS) secretion systems. All of these systems, in some cases in more than one copy, are found encoded in the genomes of all sequenced P. aeruginosa isolates (www.pseudomonas.com), with the exception of the type 4 secretion system (T4SS). This combination of secretion nanomachines is dedicated to the release of enzymes and toxins, which are involved, for example, in the degradation of complex carbon sources (2), the acquisition of iron (3), the degradation of host tissues (4, 5), the subversion of eukaryotic host cell signaling (6), and even motility (7, 8).

The T6SS of P. aeruginosa was discovered in 2006 (9). This resulted in rejuvenation of the field by bringing in novel and important concepts. It was already noteworthy that several secretion systems coevolved with machines involved in the assembly of extracellular appendages (10). For example, the type II secretion system (T2SS) is similar to the type IV pilus assembly machine (11), the type III secretion system (T3SS) has similarity with the basal body of flagella (12), and the T4SS has similarity with conjugative pili (13). In contrast, the T6SS is similar to the contractile tail of bacteriophages (14–16). One remarkable feature is the tube formed by hexameric rings of the Hcp protein (9, 17), a structural homologue of the gp19 component of the bacteriophage T4 tail tube (18). Another striking protein is VgrG, which resembles the heterotrimeric gp27_3_-gp5_3_ complex of the phage (18–21). In this complex, the gp5 protein forms a rigid helix, made of regularly spaced series of β-strands, which acts as a needle to puncture the bacterial cell envelope (22). In VgrG proteins associated with the T6SS, the C-terminal domain is similar to gp5, whereas the N terminus is similar to gp27. A further observation is the conservation in the T6SS of a sheath-like structure which is contractile and made of the gp18 protein of the T4 phage (15, 23). In the T6SS, this sheath structure is seen as long tubules by electron microscopy, but in cross section it forms cogwheel-like structures. Whereas the bacteriophage sheath contains a single protein, the T6SS counterpart is made of two interacting proteins: VipA-VipB in the case of Vibrio cholerae (24) and HsiB-HsiC in the case of P. aeruginosa (25). The T6SS is thus considered an inverted bacteriophage tail whose contraction will result in breaching of the bacterial cell envelope, allowing secretion of proteins/effectors.

Until recently, only a few T6SS substrates were described. One important example is the VgrG1 protein, which is an evolved puncturing device from V. cholerae, since it has a C-terminal extension consisting of an actin cross-linking domain (21, 26). Remarkably, this domain is translocated into the cytosol of macrophages (27, 28). While a few examples of this type were studied experimentally, it quickly became evident that one main function of the T6SS is not to subvert the host cell but to transport toxins into bacterial competitors and kill them (29). The toxin-encoding genes are not necessarily linked with the T6SS cluster, e.g., as is the case with the first P. aeruginosa T6SS (H1-T6SS) and the three associated pairs of toxin-antitoxin (Tse1 to -3 and Tsi1 to -3). These are encoded on distinct loci but are coregulated with the T6SS genes via the RetS/Gac/Rsm signaling pathway (29, 30). Since the discovery of these toxins, similar examples have been found in a number of bacterial species, including V. cholerae, Serratia marcescens, and Burkholderia species (31–34). In several cases, these toxins have been shown to degrade the peptidoglycan of the target bacterial cells, which results in rounding and lysis (35).

Whereas the *hcp* and *vgrG* genes encode core components of the T6SS machine, genomic analysis indicated that several of these genes are located distantly from any T6SS gene clusters (36). Importantly, it has been proposed that genes located downstream of the *vgrG* loci encode potential effectors, many of which are described as phospholipases (37, 38). The P. aeruginosa genome carries three T6SS clusters (39) and 10 *vgrG* genes, in the case of strain PAO1 (19). Interestingly, no *vgrG* gene could be found in the vicinity of the H2-T6SS gene cluster in the PAO1 genome. Instead, it was recently proposed that in PAO1, the H2-T6SS is involved in the secretion of a bacterial toxin with phospholipase activity (37). The toxin has been called Tle5, and the corresponding gene is found adjacent to a *vgrG* gene which is distal from the H2-T6SS locus. However, in the case of the virulent P. aeruginosa strain PA14 (40), the H2-T6SS cluster is linked with an 11th *vgrG* gene and a gene encoding a protein from the Rhs family (41). In the present study, we investigated the assembly of the PA14 H2-T6SS and whether the distinct genetic organization is relevant to the difference in behavior of the PAO1 and PA14 strains.

## MATERIALS AND METHODS

### Strains, plasmids, and growth conditions.

The strains and plasmids used in this study are described in Table 1. P. aeruginosa strains were grown in tryptone soy broth (TSB) at 37°C with agitation. Escherichia coli strains were grown in LB broth at 37°C with shaking. The pKNG101 suicide vector was used to generate deletion mutants or chromosomal insertions into the P. aeruginosa genome as described in Table 1. pCR2.1 was used as a cloning vector. pET28a and pDEST42 were used for protein overproduction. For induction of the pBAD promoters, 2% arabinose was added to the growth medium.

**TABLE 1:** Bacterial strains and plasmids used in this study

Strain or plasmid	Description	Source or reference
Strains		
Pseudomonas aeruginosa strains		
PA14	Wild-type P. aeruginosa PA14	Laboratory collection
PAO1	Wild-type P. aeruginosa PAO1, prototroph, *chl-2*	Laboratory collection
PAO1 Δ*clpV2*	PAO1 *clpV2* deletion mutant	50
PAO1 ΔH2-T6SS	PAO1 carrying a deletion of the H2-T6SS genes from mid-*hsiA2* to mid-*clpV2*	This study
PA14-DP	PA14 carrying divergent arabinose-inducible pBAD promoters upstream of *hsiA2* and *hcp2*, interspaced by a single copy of the *araC* regulator	This study
PA14-DP ΔH2	PA14-DP strain carrying a deletion of the H2-T6SS genes from mid-*hsiA2* to mid-*clpV2*	This study
PA14-DP Δ*stp2*	PA14-DP strain carrying a clean deletion of *stp2* (PA14_42890)	This study
PA14-DP *vgrG14-v5*	PA14-DP producing a V5-His_6_-tagged version of VgrG14 (PA14_43080) following the chromosomal introduction of the required sequence	This study
PA14-DP *rhsP2-v5*	PA14-DP producing a V5-His_6_-tagged version of RhsP2 (PA14_43100) following the chromosomal introduction of the required sequence	This study
PAK	Wild-type P. aeruginosa PAK	Laboratory collection
PA14::*pscC*	PA14 carrying a transposon insertion in PA14_42350 (*pscC*)	49
PA14 Δ*pscC*	PA14 with a clean deletion of PA14_42350	This study
PA14 Δ*pscC* ΔH2	PA14 with a clean deletion of PA14_42350 and deletion of the H2-T6SS cluster	This study
PA14 Δ*pscC* Δ*vgrG14-rhsP2*	PA14 Δ*pscC* strain carrying a clean deletion of the region carrying *vgrG14* and *rhsP2* (PA14_43090 and PA14_43100)	This study
PA14-DP Δ*stp2 vgrG14-v5*	PA14-DP producing a V5-His_6_-tagged version of VgrG14 in a Δ*stp2* background	This study
PA14-DP Δ*stp2 rhsP2-v5*	PA14-DP producing a V5-His_6_-tagged version of RhsP2 in a Δ*stp2* background	This study
E. coli strains		
One-Shot TOP10	Host strain for pCR2.1 derivatives	Invitrogen
CC118 λ*pir*	Host strain for pKNG101 replication	Laboratory collection
1047	Carries conjugative plasmid pRK2013 for mobilization of pKNG from CC118 into Pseudomonas spp.	62
BL21(DE3)	Host strain for pET28a derivatives	Laboratory collection
Plasmids		
pKNG ΔH2-T6SS	pKNG101 suicide vector for deletion of H2-T6SS (mid-*hsiA2* to mid-*clpV2*)	This study
pKNG Δ*vgrG14-rhsP2*	pKNG101 suicide vector for deletion of *vgrG14* to *rhsP2*	This study
pKNG Δ*pscC*	pKNG101 suicide vector for deletion of *pscC*	This study
pKNG ΔH2 promoter	pKNG101 suicide vector for deletion or insertion into the H2-T6SS promoter region	This study
pKNG Δ*vgrG14* stop codon	pKNG101 suicide vector for deletion or insertion into the stop codon of *vgrG14*	This study
pKNG Δ*rhsP2* stop codon	pKNG101 suicide vector for deletion or insertion into the stop codon of *rhsP2*	This study
pCR2.1-*araC*pBAD	Cloning vector containing the pBAD *araC* region from the arabinose-inducible pJN105 vector	63
pCR2.1-pBAD*araC*pBAD	Cloning vector containing divergent pBAD promoter regions interspaced by a single copy of the *araC* gene	This study
pCR2.1 v5-hisx6	Cloning vector containing the V5-His-_6_ sequence from pDEST42	This study
pET28a *hsiB2*	Plasmid for overexpression of His-tagged HsiB2	This study
pDEST42-*hcpA*	Plasmid for overexpression of PA1512 (HcpA), identical to Hcp2	This study

### Bioinformatic analysis.

Amino acid sequences of P. aeruginosa proteins were obtained from www.pseudomonas.com (42). Secondary structure predictions were made using the online Psipred service (43), and phylogenetic analysis was performed at www.phylogeny.fr, using the “one-click” option (44).

### Engineering arabinose-inducible H2-T6SS genes into PA14 strains.

A PA14 strain carrying inducible H2-T6SS genes was generated by the introduction of arabinose-inducible pBAD promoters and the regulatory *araC* gene at the H2-T6SS chromosomal location. This was achieved by manipulating the existing *araC* pBAD arrangement found in arabinose-inducible promoters, e.g., pJN105 (45). This promoter region was subcloned into pCR2.1, and the pBAD promoter region was amplified using primers shown in Table 2 and, following restriction digestion, inserted in the opposite orientation upstream of the *araC* gene, flanked by NheI and NdeI restriction sites.

**TABLE 2:** Cloning primers used in this work

Primer purpose and ID	Function or target	Sequence (5′–3′)
Divergent promoter engineering		
OAL771	5′ pBAD region duplication	GGAATTCCATATGTCAGAGAAGAAACCAATTGTCCATATTG
OAL772	3′ pBAD region duplication	GGAATTCCATATGAAAACGGGTATGGAGAAACAGTAGAG
OAL601	5′ region upstream of H2-T6SS promoter	GGGCCCGATCGGTACGTTCTCGT
OAL586	3′ region upstream of H2-T6SS promoter	GCAGCTAGCTTTCATATGGTTAAGATATTCATTGGCGCAC
OAL587	5′ region downstream of H2-T6SS promoter	AACCATATGAAAGCTAGCTGCGAGGGGTGGTCCAACC
OAL588	3′ region downstream of H2-T6SS promoter	ACTAGTTCGTCGGAGCCGGAG
OAL589	5′ region outside H2-T6SS promoter	CTTCAGCCACCAGGCG
OAL590	3′ region outside H2-T6SS promoter	CTGCCTGGCGCGGG
OAL591	pBAD promoter screening primer	CGCGTAACAAAAGTGTC
Cloning of V5-His_6_ region		
OAL599	5′ V5-His_6_ coding region	GCTAGCCCATTCGAAGCTTGAAGGTAAGCCTAT
OAL600	3′ V5-His_6_ coding region	GCTAGCTCAATGGTGATGGTGATGATGACC
Mutation of *vgrG14* stop codon		
OAL602	5′ region upstream of *vgrG14* stop codon	CACCAACGCCACGACACC
OAL597	3′ region upstream of *vgrG14* stop codon	TTTGCTAGCTTTGGGAGGTCACAGGCACCGTC
OAL598	5′ region downstream of *vgrG14* stop codon	CCAAAGCTAGCAAACGCCCTCTCCCCGCG
OAL603	3′ region downstream of *vgrG14* stop codon	CTTGCACTTCTCGCACTC
OAL808	5′ region outside *vgrG14* stop codon	GCTACAACGAGCTGCGCATCGAGG
OAL809	3′ region outside *vgrG14* stop codon	GCAGGTTGTCGATAGCGGTAGTCG
Mutation of *rhsP2* stop codon		
OAL1136	5′ region upstream of *rhsP2* stop codon	AATAAAACTCGCTGTCCCGAAGCATTAG
OAL1137	3′ region upstream of *rhsP2* stop codon	CATGAGTTTTCTGCTAGCGGGTCTATTACTTCGAAGTCTATTAGGTTCTTGTTG
OAL1138	5′ region downstream of *rhsP2* stop codon	GAAGTAATAGACCCGCTAGCAGAAAACTCATGAAAACCATTTATAACTTCAAACAGCG
OAL1139	3′ region downstream of *rhsP2* stop codon	TTCATTTGCTTCTGTCTTTTGGTTTTTTATATAACC
OAL1160	5′ region outside *rhsP2* stop codon	CGGGACGCTACCTGACCCCC
OAL1161	3′ region outside *rhsP2* stop codon	GATGCGTGAACTTCCTGAGCCAGG
*hsiB2* cloning into pET28a		
OAL1171	5′ *hsiB2*	GCCATATGGCCAAAGAAGGCTCGGTAGCC
OAL1172	3′ *hsiB2*	GCCTCGAGGGCGTCCTGGGAGGGGGC
Deletion of *pscC*		
OAL1389	5′ region upstream of *pscC*	ACGCAACCTGTGCCAGGCACAGG
OAL1399	3′ region upstream of *pscC*	CTAATTCCCGCGGCGCATCAGGGACGCC
OAL1400	5′ region downstream of *pscC*	ATGCGCCGCGGGAATTAGCATGGCCTGGAAGATCC
OAL1401	3′ region downstream of *pscC*	TAGGCGCGCACCCTCGCC
OAL1484	5′ region outside *pscC*	GAGGTCCTCGAATGCCTCTGG
OAL1485	3′ region outside *pscC*	TTGTCCTCCAGGTAGCCGCC
Deletion of H2-T6SS (mid-*hsiA2* to mid-*clpV2*)		
OAL996	5′ region upstream of H2-T6SS	GACTGGTTGAAAATCCTGGAAAAC
OAL997	3′ region upstream of H2-T6SS	TCAGGCGAACGGCCTCCTGCTGGGCGC
OAL998	5′ region downstream of H2-T6SS	AGGAGGCCGTTCGCCTGAGGTGGGTGC
OAL999	3′ region downstream of H2-T6SS	CAACACGGTATAGGGGTTGTG
OAL1000	5′ region outside H2-T6SS	GAATTGTTAAGATATTCATTGGCGCAC
OAL1001	3′ region outside H2-T6SS	TCGAGCAGCAGGGTTCCGCCATCCGCG
Deletion of *vgrG14-rhsP2*		
OAL1900	5′ region upstream of *vgrG14-rhsP2*	AGGTCTTCGACAAGGCCTCGCCG
OAL1901	3′ region upstream of *vgrG14-rhsP2*	TGTTTGAAGCCGTTGTCCCTCACTGGCGCAG
OAL1902	5′ region downstream of *vgrG14-rhsP2*	GGACAACGGCTTCAAACAGCGTATCAAAGAAGACCCCG
OAL1903	3′ region downstream of *vgrG14-rhsP2*	TATGATTCCCATGGATAGGGGGTTTTCATTTGC
OAL1984	5′ region outside *vgrG14-rhsP2*	TACCAGGAAGGCCACGAG
OAL1985	3′ region outside *vgrG14-rhsP2*	CCCATGGATAGGGGGTTT
Deletion of *stp2*		
OAL346	5′ region upstream of *stp2*	CTTCTTCGAAACCTACATGCG
OAL347	3′ region upstream of *stp2*	TCATTGGCTGCGTTGCATCAGAGCTGC
OAL348	5′ region downstream of *stp2*	ATGCAACGCAGCCAATGAACGAACCGC
OAL349	3′ region downstream of *stp2*	CGTGGACGTAGGCCAGAA
OAL350	5′ region outside *stp2*	TACCTGTATCTCAACCAGCGC
OAL351	3′ region outside *stp2*	CAAGCGGGACAAGATGTTAAT

To manipulate the H2-T6SS promoter region, 500-bp regions upstream and downstream of the intergenic region were cloned using primers defined in Table 2. A total of 50 bp upstream of the *hsiA2* and *hcp2* open reading frames were conserved. The 500-bp regions were fused by overlap extension PCR, incorporating NheI and NdeI restriction sites into the overlap region. This generated an ∼1-kb mutator fragment, which was cloned into the pCR2.1 cloning vector.

This mutator plasmid was subsequently modified by introduction of the divergent pBAD construct within the overlap region by restriction digestion. This construct was confirmed by PCR, subcloned into the pKNG101 suicide vector, maintained in E. coli CC118 λ*pir*, and mobilized into P. aeruginosa by three-partner conjugation. Double-recombination events resulting in the exchange of the native promoter region with the divergent promoter construct were selected on sucrose plates, generating the PA14-DP (divergent promoter) strain, and confirmed by PCR using external primers and an internal primer specific to the pBAD region (Table 2).

### Construction of clean deletion mutants.

Deletion mutants were generated in P. aeruginosa by using the pKNG101 suicide vector as previously described (46). Primers used are described in Table 2. Deletion of the H2-T6SS cluster in P. aeruginosa PA14 and PAO1 was achieved by deletion of a region spanning from mid-*hsiA2* to mid-*clpV2*. Deletion of *pscC* was achieved by deletion of the PA14_43250 open reading frame. The *vgrG14* to *rhsP2* cluster was disrupted by deleting a region spanning from the beginning of *vgrG14* (PA14_43080) to the end of *rhsP2* (PA14_43100), including the PA14_43090 open reading frame. pKNG mutator plasmids were maintained in E. coli CC118 λ*pir* and mobilized into P. aeruginosa by three-partner conjugation. Double-recombination events resulting in the deletion of the required chromosomal regions were selected on sucrose plates and verified by PCR using external primers (Table 2).

### Construction of PA14 strain chromosomally encoding V5-tagged VgrG14 and RhsP2 proteins.

The PA14-DP strain was engineered to allow production of a C-terminally V5-tagged version of either VgrG14 or RhsP2 to allow detection of the protein. This was achieved by exchange of the native stop codon of either *vgrG14* or *rhsP2* with the sequence encoding the V5 and His_6_ tags, followed by a new stop codon. The sequence encoding the V5-His_6_-stop sequence was amplified from pDEST42, flanked by restriction sites engineered into primers described in Table 2, and cloned into the pCR2.1 vector.

To disrupt the desired chromosomal location, 500-bp regions upstream and downstream of the native stop codon of the gene of interest were amplified using primers defined in Table 2 and fused by overlap extension PCR, incorporating a restriction site into the overlap region. This generated the mutator fragment, which was cloned into the pCR2.1 cloning vector.

The mutator fragment was subsequently modified by insertion of the V5-His_6_-stop sequence between the two 500-bp regions by restriction digestion. The modified mutator fragment was subcloned into the pKNG101 suicide vector, maintained in E. coli CC118 λ*pir*, and mobilized into P. aeruginosa by three-partner conjugation. Double-recombination events resulting in the insertion of the sequence encoding the V5-His_6_ tag at the stop codon of the required gene were selected on sucrose plates and verified by PCR.

### qRT-PCR analysis.

Overnight cultures were subcultured in TSB, grown to mid-exponential phase, and harvested into RNAlater (Ambion). RNA extraction, reverse transcription (RT), and quantitative RT-PCR (qRT-PCR) were carried out as previously described (47). The primers used for amplification are shown in Table 3. Gene expression was normalized to expression of *rpoD* and expressed as a ratio relative to the value for the PA14 wild-type strain, which was set to 1.

**TABLE 3:** qPCR primers used in this work

Primer ID	Direction	Target gene	Sequence (5′–3′)
OAL920	Forward	*stk2*	CCGCTGGTAGCATTGAAGCT
OAL921	Reverse	*stk2*	GGCGAACTCGCTATAGAGCAA
OAL922	Forward	*hsiG2*	GTGTTCGCTTCGGTTCTGAAC
OAL923	Reverse	*hsiG2*	GTGCTCCTCACCCGCAACT
OAL924	Forward	*hsiF2*	ACTACGGGTTGCCCGATCTC
OAL925	Reverse	*hsiF2*	GGTTCGTAAGCCTCGATGAAAC
OAL926	Forward	PA14_43090	GCCGTCACCTGCTACCGATA
OAL927	Reverse	PA14_43090	GATCAGGTAACGGCCGAACA
OAL928	Forward	*rhsP2*	GACAAGGACGCCAACATCCT
OAL929	Reverse	*rhsP2*	CGAAAGTATGCAGCAGTTTCAGTT
OAL511	Forward	*hsiA2*	GGTTGACCTGGGCCCTCTAC
OAL512	Reverse	*hsiA2*	GATGGATCTCGACCCAATGC
OAL540	Forward	*hcp2/A/B/C*	CCAAGGTCGAGATCCAGTGGTA
OAL541	Reverse	*hcp2/A/B/C*	GTAGTCCTTGATGTCGACGATGAT
OAL542	Forward	*vgrG14*	TCACCCCGGCCCAGAT
OAL543	Reverse	*vgrG14*	TCTCGCACTCTTCGCAGAAG
OAL820	Forward	*rpoD*	AGGCCGTGAGCAGGGATAC
OAL821	Reverse	*rpoD*	TCCCCATGTCGTTGATCATG
OAL538	Forward	*vgrG5*	GCCCGAAGGGTGAGGAA
OAL539	Reverse	*vgrG5*	CTCGCGATCCCAGTGGAAT
OAL536	Forward	*vgrG2b*	GGAGCCGGGAAAGACGTT
OAL537	Reverse	*vgrG2b*	AGGCTTCCCCGAACTCGTT
OAL721	Forward	*pelA*	CCTTCAGCCATCCGTTCTTCT
OAL722	Reverse	*pelA*	TCGCGTACGAAGTCGACCTT
OAL822	Forward	*tse3*	GGCACGCAATGCCTTGAT
OAL823	Reverse	*tse3*	GCAGATGTCGAAGAAGGTGATG

### Production of antibodies against Hcp2 and HsiB2.

The gene encoding an *hcp2* homolog (PA1512) was transferred by LR recombination (Invitrogen) from the pDONR shuttle vector to the pETDEST-42 expression vector. *hsiB2* was cloned into pET28a by using primers described in Table 2.

For each construct, protein expression was induced at an optical density at 600 nm (OD_600_) of 0.6 by addition of 0.5 mM isopropyl-β-d-thiogalactopyranoside. Following induction, cultures were incubated at 18°C overnight with agitation. Cells were harvested and resuspended in 50 mM HEPES, 500 mM NaCl, 200 nM imidazole, and Complete EDTA-free protease inhibitors, pH 7.2, and then lysed by use of a French press. The lysate was centrifuged at 3,000 × *g* for 15 min, and the supernatant was applied to His-trap columns (GE Health Care) for purification by use of imidazole gradients. Protein was subsequently purified by size-exclusion chromatography under final buffer conditions of 50 mM Tris, 250 mM NaCl, pH 8. Purified protein was concentrated and sent for antibody production in rabbits (Eurogentec).

### Protein secretion assay.

T6SS secretion assays were performed following growth with or without 2% arabinose in the culture medium to induce the arabinose promoters. Culture supernatants were prepared as previously described (19) and resuspended to 0.1 OD_600_ unit per 10 μl of Laemmli buffer. Cell extracts were prepared by harvesting 1 ml of bacterial culture by centrifugation and were resuspended to 0.01 OD_600_ unit per 10 μl of Laemmli buffer. Samples were analyzed by SDS-PAGE and Western blotting.

T3SS secretion assays were performed as described for T6SS secretion, but calcium chelation was used to induce the T3SS machinery. This was achieved by supplementing cultures with 5 mM EGTA and 20 mM MgCl_2_ prior to incubation, as previously described (48).

### SDS-PAGE and Western blotting.

Cell extracts and supernatant samples were boiled at 95°C for 10 min prior to separation by SDS-PAGE. Cell extracts were loaded at an equivalent of 0.1 OD_600_ unit per well, and supernatants at 1.0 OD_600_ unit per well. Following electrophoresis, proteins were transferred to nitrocellulose membranes.

Antibodies against Hcp2 and HsiB2 were generated as described above and used at a 1:1,000 dilution. Anti-RNA polymerase (Neoclone) was used at a dilution of 1:10,000. Anti-V5 antibody (Invitrogen) was used at a dilution of 1:5,000. Anti-PcrV antibody was used at 1:1,000. Primary antibodies were incubated for 1 to 2 h at room temperature, followed by 45 min of incubation with the appropriate secondary antibody (goat anti-rabbit–horseradish peroxidase [HRP] or rabbit anti-mouse–HRP) at a dilution of 1:5,000. Western blots were developed with SuperSignal West Pico chemiluminescent substrate (Pierce/Thermo Scientific) and visualized using a Las3000 Fuji imager.

### Cytotoxicity on RAW macrophages monitored by LDH release assay.

The cytotoxicity of the parental strain PA14 and its isogenic Δ*pscC* and Δ*pscC* Δ*H2-T6SS* mutants was compared to the toxicity of the PA14::*pscC* transposon mutant (49) using the murine RAW 264.7 macrophage cell line (ATCC TIB-71). Raw macrophages were routinely grown in Dulbecco modified Eagle medium, GlutaMAX I, sodium pyruvate, and phenol red (Gibco) supplemented with 10% fetal bovine serum (Gibco) and nonessential amino acids (Gibco). Macrophages were grown to 80% confluence in 96-well plates (BD Biosciences), washed with sterile phosphate-buffered saline (PBS), and incubated for 1 h prior to infection with RPMI 1640 without phenol red. Macrophages were infected with late-exponential-phase P. aeruginosa at a multiplicity of infection of 10 for 3 h at 37°C in the presence of 5% CO_2_. The macrophage infection was synchronized by pelleting the bacteria with a 5-min centrifugation step at 200 × *g.* After the infection time point, the plates were centrifuged again for 5 min at 200 × *g* to sediment cell debris and bacteria prior to supernatant collection. The release of cytosolic lactate dehydrogenase (LDH) into the culture supernatant was measured with the CytoTox96 nonradioactive cytotoxicity assay (Promega). Cytotoxicity was calculated relative to that of noninfected cells, set at 0%, and that of cells lysed with 1% Triton X-100, which was set at 100%.

### Gentamicin protection assay and P. aeruginosa internalization in HeLa cells.

P. aeruginosa internalization was assessed using the gentamicin protection assay as previously described (50), with the exception that antibiotic treatment of extracellular bacteria was performed for 75 min. The PA14-DP Δ*pscC* strain was grown in the presence of 2% arabinose. Arabinose (0.2%) was also added to HeLa cell culture medium during the course of the infection with the PA14-DP Δ*pscC* strain. Paired *t* tests were performed using Excel (Microsoft).

## RESULTS

### Comparison of P. aeruginosa H2-T6SS clusters.

**FIG 1:**
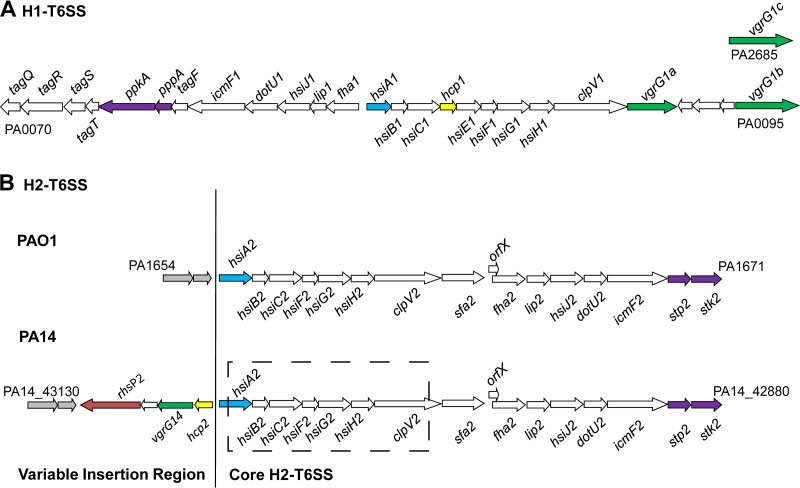
Organization of the H1- and H2-T6SS clusters in P. aeruginosa. The H1-T6SS cluster (A) and two variations of the H2-T6SS cluster, from PAO1 and PA14 (B), are shown for comparison. Arrows represent the orientations of open reading frames. *hsiA*, highlighted in blue, is conserved across the H1- and H2-T6SSs, as are the majority of the core T6SS genes (white arrows). Two genes encoding key structural components of the T6SS are shown in green (*vgrG*) and yellow (*hcp*). *rhsP2*, a gene specific to the H2-T6SS from PA14, is shown in red. Genes encoding serine-threonine kinases/phosphatases are shown in purple. These are known to be involved in posttranslational control of the H1-T6SS (*ppkA* and *pppA*) and are conserved in the H2-T6SS (*stp2* and *stk2*). Genes not related to the T6SS at the boundary of the H2-T6SS cluster are shaded in gray. The core H2-T6SS cluster is conserved in PAO1 and PA14, but four additional genes which are absent in PAO1 are present in the PA14 genome, at the location indicated by the vertical line. The hatched box indicates the region removed in the construction of a Δ*H2-T6SS* mutant strain. PA numbers are included for the first and last genes in each cluster.

A growing number of P. aeruginosa isolates have had their genomes sequenced in the past few years. Eleven such genomes are available for comparison at www.pseudomonas.com. For each of these genomes, the three known P. aeruginosa T6SS clusters could readily be identified. Interestingly, it has been reported previously that the H2-T6SS from PAO1 is not physically linked to any *hcp* or *vgrG* genes (51). However, on the PA14 genome, an *hcp* gene and a *vgrG* gene are located next to *hsiA2* and transcribed in a divergent orientation (Fig. 1). Here these genes are called *hcp2* (indicating that it is linked with the H2-T6SS) and *vgrG14* (indicating that it has been found in PA14), respectively. These two genes appear to be organized in an operon with two other genes, namely, PA14_43090 and PA14_43100. Whereas BLAST analysis did not retrieve any homologous protein of known function for PA14_43090, PA14_43100 encodes a protein belonging to the Rhs (recombination hot spot) family (41, 52), which we named RhsP2 (for Rhs Pseudomonas 2) (Fig. 1). In addition to PA14, only two other P. aeruginosa genomes display a similar organization, namely, those from the PA39016 and NCGM2.S1 strains. In the case of NCGM2.S1, which is a highly multidrug-resistant strain (53), the *rhsP2* gene is linked with a *vgrG* gene and an *hcp* gene as in PA14 (Fig. 1). In contrast, for P. aeruginosa PA39016, no *hcp* or *vgrG* gene was found at this location (data not shown). Finally, two genes, identified as PA1654 and PA1655, are found upstream of *hsiA2* on the PAO1 genome and encode a putative aminotransferase and glutathione *S*-transferase, respectively (Fig. 1). In PA14, PA39016, and NCGM2.S1, these genes are found downstream of the *rhsP2*-containing cluster, suggesting that this cluster has been lost in PAO1 or was acquired and inserted at this position in the above 3 strains.

### Activation of H2-T6SS gene expression.

**FIG 2:**
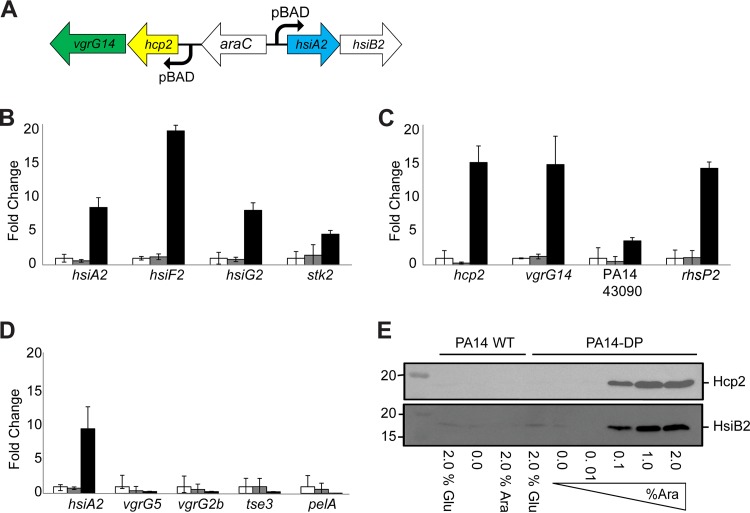
Artificial induction of the H2-T6SS through the introduction of arabinose-inducible promoters. (A) The H2-T6SS promoter region was replaced with two divergently acting pBAD promoters (curved arrows) and a single copy of the *araC* regulatory gene. Following two events of homologous recombination of a suitably designed mutator plasmid, using the pKNG suicide vector, the PA14-DP (divergent promoter) strain was generated. (B to D) The activity of the promoters was tested by qRT-PCR. The expression of various H2-T6SS genes was tested in wild-type PA14 (white bars), uninduced PA14-DP (gray bars), and PA14-DP induced with 2% arabinose (black bars). Fold changes of gene expression in the induced or uninduced divergent promoter strain are shown relative to the expression in the PA14 wild-type strain, which was set at 1.0. (B) Expression of genes carried in the core PA14 H2-T6SS cluster; (C) expression of genes carried adjacent to the H2-T6SS cluster in PA14; (D) expression of genes carried outside the H2-T6SS cluster, including replication of the expression of *hsiA2* as a positive control. Error bars show the standard deviations for three replicates. (E) Western blot analysis of the production of H2-T6SS proteins from whole-cell extracts of PA14 and the PA14-DP derivative. Blots were probed with anti-Hcp2 (top) or anti-HsiB2 (bottom), and the expected sizes of these proteins are indicated to the right of the blots. The strains tested are indicated above the blots, while concentrations of glucose (Glu) and arabinose (Ara), used to repress and induce the pBAD promoters, respectively, are indicated below the blots. Molecular size markers are indicated to the left. WT, wild type.

Expression of the H2-T6SS cluster in PAO1 has been reported to be dependent on various regulatory elements, such as iron depletion and quorum sensing (50, 51). However, expression is rather low and not constitutive. We aimed at engineering a strain in which the expression of the H2-T6SS genes could be tightly controlled to ease the study of the system under laboratory conditions. Since the PA14 H2-T6SS gene cluster is organized in two transcriptionally divergent units, we constructed a strain with two inducible arabinose promoters (pBAD) inserted in divergent orientation within the intergenic region, yielding the PA14-DP strain, as described in Materials and Methods (Fig. 2A and Table 1). Using this strain, qRT-PCR experiments were performed in the presence or absence of the arabinose inducer and probed the expression of genes in the two divergent transcription units (Fig. 2). Addition of arabinose drastically increased (20-fold) expression of the *hsiF2* gene, and expression of the last gene in the cluster, *stk2*, was still upregulated 5-fold (Fig. 2B). In other cases, such as with *hsiA2* or *hsiG2*, increased expression was about 10-fold higher (Fig. 2B). Furthermore, in the divergent transcription unit, *hcp2*, *vgrG14*, and *rhsP2* were increased about 15-fold, while PA14_43090 displayed <5-fold upregulation (Fig. 2C). Finally, expression of genes located outside the H2-T6SS cluster was not affected by arabinose addition (Fig. 2D).

### Production of the H2-T6SS machine.

In order to evaluate whether arabinose-dependent induction of the H2-T6SS genes effectively resulted in protein production, antibodies against two components of the H2-T6SS system, HsiB2 and Hcp2, were generated. PA14 and the PA14-DP derivative were grown in LB medium containing increasing concentrations of arabinose, i.e., 0, 0.01, 0.1, 1, and 2%. Cell extracts were prepared and proteins separated by SDS-PAGE, followed by Western blot analysis (Fig. 2E). HsiB2 and Hcp2 could readily be detected with 0.1% arabinose induction and increased slightly at 1 and 2% concentrations of the inducer. In contrast, in the parental PA14 strain, no HsiB2 or Hcp2 bands could be detected, even in the presence of 2% arabinose.

**FIG 3:**
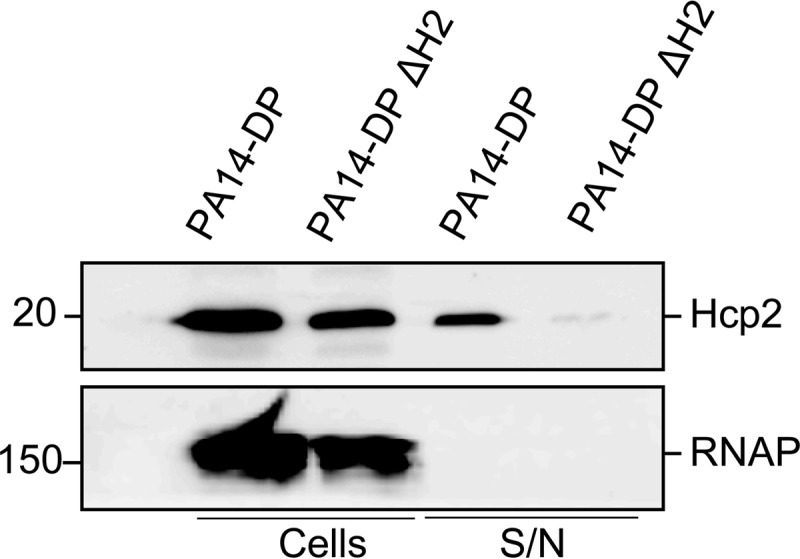
H2-T6SS-dependent secretion of Hcp2. Western blot analysis was performed on whole-cell extracts (cells) and supernatants (S/N) from PA14-DP and PA14-DP with a clean deletion in the H2-T6SS cluster (PA14-DP ΔH2; the hatched box in Fig. 1 indicates the region deleted in this strain). Blots were probed with either anti-Hcp2 (top) or anti-RNA polymerase (RNAP) (bottom) antibody, and the expected positions of these proteins are indicated on the right. The strains tested are indicated at the top, and the sample types are indicated below the blots. Molecular size standards are indicated on the left.

For other T6SSs, it is proposed that upon assembly of the system, the associated Hcp protein forms a tubule-like structure that emerges at the cell surface (9, 17, 54). It was also demonstrated that the Hcp protein could be recovered in the extracellular medium and was secreted in a T6SS-dependent manner. In order to assess whether production of the H2-T6SS machinery could be induced upon arabinose addition, it was tested whether Hcp2 could be recovered in the supernatant fraction. PA14-DP was grown in the presence of 2% arabinose to late exponential phase, and cells and supernatant were separated by centrifugation as described above. Whereas Hcp2 was largely detectable in the cell fraction, a significant proportion was also released into the medium (Fig. 3). A mutation in the H2-T6SS cluster of PA14-DP was then engineered to remove the DNA region carrying the *hsiA2* gene down to the *clpV2* gene, yielding PA14-DP ΔH2 (Fig. 1). In this case, whereas Hcp2 was still detected in the cells, the protein was absent from the supernatant fraction, indicating that its secretion is dependent on the H2-T6SS (Fig. 3).

### Characterization of the H2-T6SS *vgrG*-like gene.

**FIG 4:**
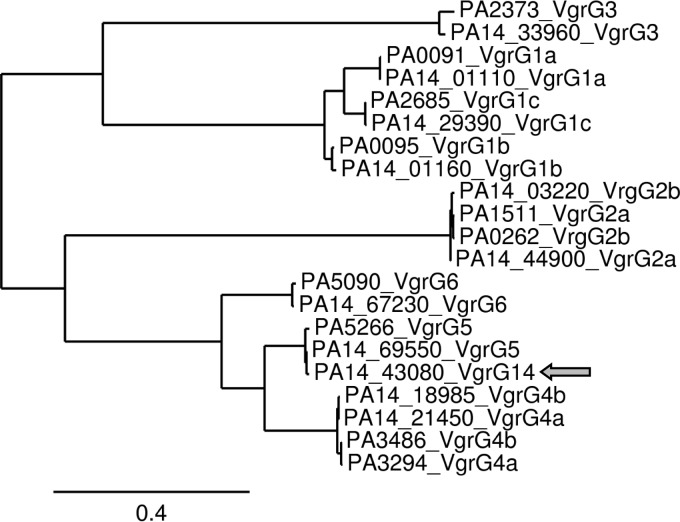
Phylogenetic analysis of P. aeruginosa VgrG proteins. VgrG amino acid sequences from P. aeruginosa PAO1 and PA14 were compared at www.phylogeny.fr. A graphical representation of the inferred tree is shown. The position of the PA14-specific VgrG14 protein is indicated by the gray arrow.

The VgrG proteins have been proposed to form the puncturing device of the T6SS apparatus (21). The C-terminal end is a repeat of β-strands whose assembly into a trimer forms a needle-like structure (19, 21). This type of VgrG protein is called “canonical VgrG.” In some cases, the series of β-strands is followed by a large extension, such as with VgrG1 or VgrG3 in Vibrio cholerae, which appears to be the effector that is transported by the T6SS (21, 27, 55). This type of VgrG protein is called “evolved VgrG.” In total, there are 10 VgrG proteins encoded on the PAO1 genome (Fig. 4) (19), among which 1 could be identified as an evolved VgrG protein (PA0262 or VgrG2b) (21). All of these *vgrG* genes are also found in the PA14 genome (49), which thus has 11 copies, considering the additional (*vgrG14*) gene. Phylogenetic analysis suggests that VgrG proteins can be classified into groups/families (19). Here we predict that VgrG14 is tightly related to another VgrG, encoded by PA5266 (VgrG5), in PAO1 (Fig. 4). Both VgrG14 and VgrG5 are predicted to be canonical proteins, with no significant C-terminal extension following the stretch of β-strands constituting the gp5 domain (data not shown). Interestingly, VgrG5 is encoded within a gene cluster that does not include any other core T6SS genes but has an *hcp* gene (PA5267) and another downstream gene (PA5265), which encodes a protein of unknown function. Genes located downstream of *vgrG* genes, even though not clustered with other T6SS genes, have been predicted to encode potential T6SS substrates (36). For example, this is the case for PA3487, encoding a phospholipase named Tle5 (called VgrG4b here) (37), which is located next to PA3486. VgrG4b is also phylogenetically related to VgrG14, whereas the canonical VgrG1a, VgrG1b, and VgrG1c proteins, which are connected to the H1-T6SS, are more distant (19) (Fig. 4). We thus predict that VgrG14 and RhsP2 could be potential substrates for the H2-T6SS.

### Analysis of VgrG14 and RhsP2 secretion.

**FIG 5:**
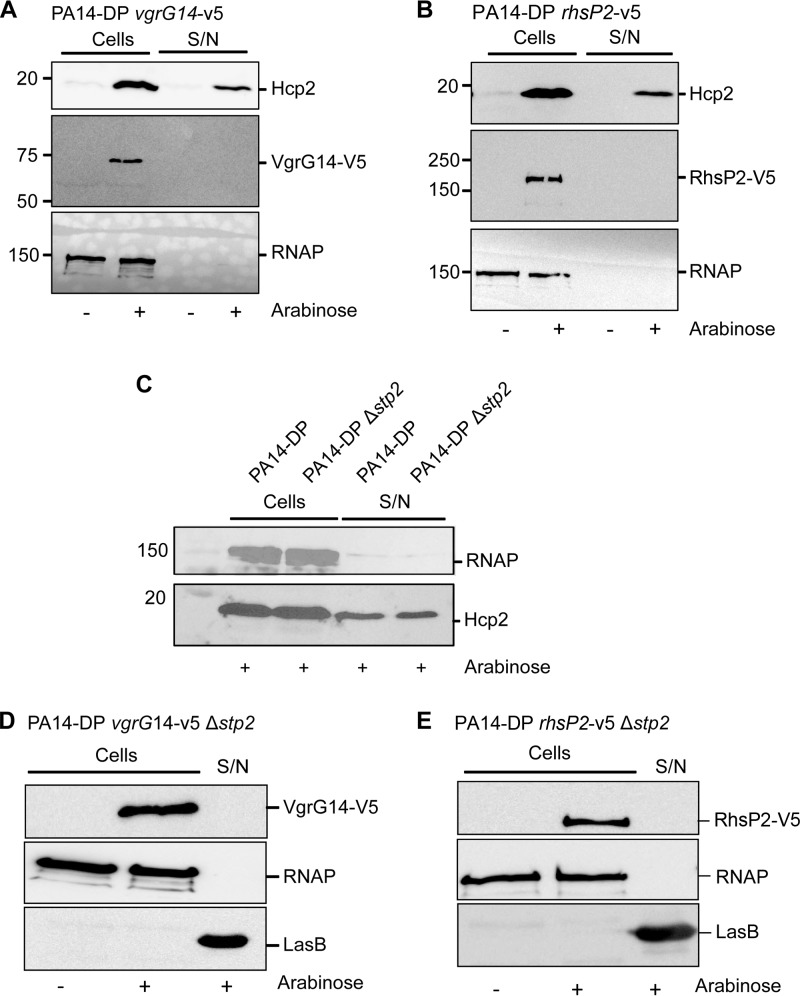
V5-tagged VgrG14 and RhsP2 proteins are produced but not secreted. Western blots were performed on whole-cell extracts (cells) and supernatants (S/N) of the indicated strains. (A and B) PA14-DP strains carrying chromosomally tagged versions of *vgrG14* (A) and *rhsP2* (B). Upper blots were probed with anti-Hcp2, central blots with anti-V5, and lower blots with anti-Hcp2. (C) Effect of *stp2* deletion on Hcp2 secretion. Western blotting was performed on PA14-DP and the isogenic PA14-DP Δ*stp2* mutant; the upper blot was probed with anti-RNAP and the lower blot with anti-Hcp2. (D and E) Western blots performed on PA14-DP Δ*stp2* strains carrying a chromosomally tagged version of either *vgrG14* (D) or *rhsP2* (E). Upper blots were probed with anti-V5 antibody, central blots with anti-RNAP antibody, and lower blots with anti-LasB (secreted control). Molecular size standards are indicated to the left, and the expected position of each protein is indicated on the right. The presence or absence of arabinose is indicated below each blot.

As for the Hcp proteins, VgrG proteins are released into the extracellular medium in a T6SS-dependent manner (19, 21). The fate of VgrG14 was assessed by engineering, in PA14-DP, a chromosomal *vgrG14* gene encoding a V5-tagged version of the protein as described in Materials and Methods. Upon addition of arabinose, expression of the H2-T6SS genes was induced, as seen by the production of Hcp2 (Fig. 5A to C). The fate of VgrG14 was then followed by Western blotting using an anti-V5 antibody. Whereas Hcp2 clearly appeared in the supernatant, VgrG14 was seen only in the cell fraction, indicating that it was not secreted in detectable amounts (Fig. 5A). Note that production of both Hcp2 and VgrG14 was strictly dependent on addition of arabinose to the growth medium.

Only a few effectors have been characterized for T6SSs that have been studied so far, and these are mainly bacterial toxins (33). However, it was recently reported that a P. aeruginosa strain produces an RhsT protein associated with virulence and the inflammatory response, although its secretion was not clearly analyzed (56). We therefore investigated whether RhsP2 could be an H2-T6SS effector. Following a strategy similar to the one described for VgrG14, a PA14-DP strain encoding a V5-tagged version of RhsP2 was engineered. Whereas the tagged protein could readily be detected in the cell fraction upon addition of arabinose, no RhsP2 could be seen in the extracellular medium (Fig. 5B).

It has been described previously that the H1-T6SS can be assembled while not actively secreting substrate (57). The activation of the system relies on the antagonistic activity of two proteins: a serine-threonine kinase (Stk) and a serine-threonine phosphatase (Stp). The kinase promotes activity via phosphorylation of the Fha protein, whereas Stp inhibits the activity of the system by dephosphorylating Fha. As for the H1-T6SS, the H2-T6SS cluster carries *fha*, *stk*, and *stp* genes (Fig. 1), which are called *fha2*, *stk2*, and *stp2* here to acknowledge their link with the H2-T6SS cluster. In order to investigate whether the H2-T6SS can be made secretion competent, an *stp2* deletion was engineered into the PA14-DP strain. We confirmed that a mutation in *stp2* did not affect the secretion of Hcp2 (Fig. 5C). However, in this case, secretion of V5-tagged VgrG14 or RhsP2 was also not detectable (Fig. 5D and E).

### Does H2-T6SS induction influence internalization in nonphagocytic cells?

Although neither Vrg14 nor RhsP2 could clearly be identified as an H2-T6SS substrate, we investigated whether the induction of the entire H2-T6SS in P. aeruginosa PA14 resulted in an observable phenotype, such as previously reported for PAO1 (50). In that case, it was shown that the H2-T6SS influences internalization in nonphagocytic epithelial cells. In order to investigate this aspect in PA14, a deletion of the *pscC* gene was engineered to inactivate the T3SS (PA14 Δ*pscC*). The existence of the T3SS effector ExoU, encoded on the pathogenicity island PAPI-1 of strain PA14 (40), renders this strain highly cytotoxic. We checked that the *pscC* mutation inactivated the T3SS and the extracellular release of PcrV (58), the T3SS tip component. Bacteria were grown under Ca^2+^-chelating conditions, and cells and supernatant were then separated by centrifugation and analyzed by Western blotting using anti-PcrV. Our data showed that in the *pscC* mutant, PcrV was no longer detectable in the supernatant (see Fig. S1A in the supplemental material). A cytotoxicity assay was also performed using RAW macrophages and monitoring LDH release. Upon introduction of the *pscC* mutation, PA14 cytotoxicity was totally abrogated, and no contribution of the H2-T6SS could be seen, since introduction of the sole H2-T6SS mutation did not alter the cytotoxic profile (see Fig. S1B).

**FIG 6:**
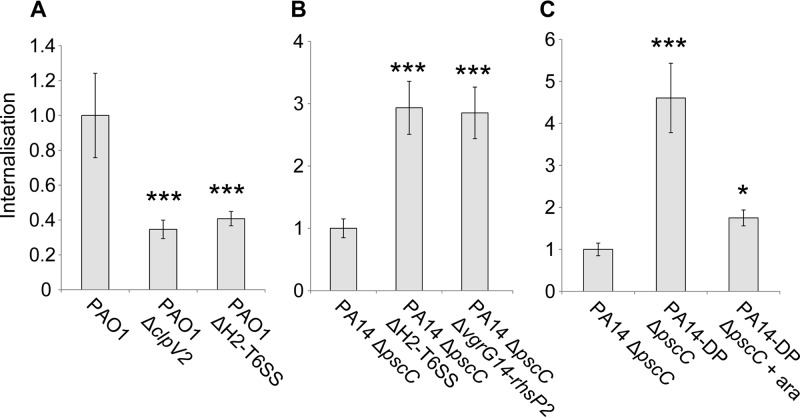
The H2-T6SS of PA14 inhibits bacterial internalization in HeLa cells. (A) Relative internalization in HeLa cells of the isogenic mutant strains PAO1 Δ*clpV2* and PAO1 lacking the core H2-T6SS cluster, including *clpV2* (PAO1 ΔH2-T6SS), compared to that of the parental strain PAO1. (B) Relative internalization of the noncytotoxic parental strain (PA14 Δ*pscC*) compared to that of isogenic mutants carrying deletions in the H2-T6SS cluster (PA414 Δ*pscC* ΔH2-T6SS) or deletion of the *vgrG14-rhsP2* region (PA14 Δ*pscC* Δ*vgrG14-rhsP2*). (C) The relative internalization of a noncytotoxic PA14 strain carrying an arabinose-inducible H2-T6SS (PA14-DP Δ*pscC*) is shown with and without arabinose induction (+ara) and compared to the internalization of the parental strain, PA14 Δ*pscC*. In all cases, the internalization of mutants is shown relative to the level exhibited by the corresponding parental strain (set to 1.0). The strain tested is indicated below each bar. Error bars show standard deviations, and the results shown are representative of three different experiments. Statistically significant differences compared to the corresponding parental strain are indicated as follows: *, *P* < 0.05; and ***, *P* < 0.01 (Student's *t* test).

A gentamicin protection assay was then performed as described previously, by monitoring the number of bacteria internalized in HeLa cells via counting the number of CFU. Using the PAO1 and PAO1 Δ*clpV2* strains, we confirmed previously published data (50) and showed that the H2-T6SS mutant had reduced (about 2-fold) internalization capability (Fig. 6A). We also engineered a mutant lacking most of the H2-T6SS gene cluster, PAO1 Δ*H2-T6SS*, and similar results were obtained (Fig. 6A). We then compared the internalization phenotypes of the PA14 Δ*pscC* strain and the H2-T6SS mutant derivative PA14 Δ*pscC* Δ*H2-T6SS*. In this case, and in contrast with the PAO1 situation, an increased level of internalization was observed in the absence of the H2-T6SS cluster (Fig. 6B), suggesting that the H2-T6SS prevents internalization. In this genetic context, there is no arabinose-inducible promoter, and the phenotype observed in PA14 may simply account for the induction of the H2-T6SS genes upon contact with epithelial cells. This is supported by the observation that with the PA14-DP Δ*psc* strain, in which the original promoter was replaced by the pBAD promoter, the internalization level was similar to that observed with the H2-T6SS mutant PA14 Δ*pscC* Δ*H2-T6SS*. It suggests that, in PA14, the original promoter is needed to activate H2-T6SS genes when bacteria are in contact with epithelial cells and thus to prevent internalization in an H2-T6SS-dependent manner. Moreover, with PA14-DP Δ*psc*, addition of arabinose drastically reduced the level of internalization (Fig. 6C), confirming that induction of the H2-T6SS prevents internalization. We concluded that the H2-T6SS in PA14 may have a different role in bacterium-host interaction and that this may be due to the use of a different panel of effectors, as suggested by the difference in H2-T6SS cluster organization observed between the PAO1 and PA14 strains. Interestingly, when we used a mutant deleted for the *vgrG14* and *rhsP2* genes in the divergent transcriptional unit (PA14 Δ*pscC* Δ*vgrG14-rhsP2*) instead of using the H2-T6SS mutant (PA14 Δ*pscC* Δ*H2-T6SS*), a similar internalization phenotype was observed (Fig. 6B), thus supporting the hypothesis that the observed H2-T6SS-dependent phenotype is also VgrG/Rhs dependent.

## DISCUSSION

Previous studies have shown that the H2-T6SS from P. aeruginosa PAO1 can be induced under low-iron conditions or during the growth transition from planktonic to stationary growth, in a quorum sensing-dependent manner (50, 51). In the present study, we used another strain, PA14, and engineered a pBAD promoter into the chromosome, which resulted in tightly controllable expression of the H2-T6SS genes. The addition of arabinose resulted not only in transcriptional upregulation of the gene cluster but also in production of T6SS components, as seen with HsiB2.

The lack of *hcp* and *vgrG* genes linked to the PAO1 H2-T6SS genes is puzzling and suggests that independent clusters could be associated with the H2-T6SS to provide the missing *hcp* and *vgrG* genes, which are described as essential for T6SS function. In this respect, several reports have proposed that the *vgrG2a* and *vgrG2b* genes, both linked to an *hcp* gene, could potentially be associated with the H2 system in PAO1 (19, 36), though no data have yet supported this hypothesis. In contrast, analysis of the PA14 genome revealed that next to the genes encoding the H2-T6SS core components, and transcribed divergently, a cluster of 4 genes is found and encodes an Hcp protein (Hcp2), a VgrG protein (VgrG14), and two other proteins, one of which (PA14_43100 protein) is a putative Rhs protein (RhsP2). Interestingly, the *hcp* gene encodes a protein which is 100% identical to all other Hcp proteins encoded from the so-called *vgrG* islands (19), which are not linked to a T6SS cluster (36, 37, 55). The sequence conservation with T6SS-linked Hcp proteins, such as Hcp1, is weaker. However, the crystal structure of an Hcp protein of this subfamily shows a similar hexameric structure and only a slightly different organization in the Hcp ring-containing nanotubes, which are stacked head to head instead of head to tail (54). Our study showed that artificial but simultaneous induction of the two gene clusters in PA14 resulted in the H2-T6SS-dependent secretion of Hcp2. This observation demonstrates that the two sets of genes are functionally linked and that VgrG14 and RhsP2 are obvious candidate H2-T6SS substrates.

VgrG proteins are definitely part of the T6SS machinery, but in some cases they have been shown to have dual functions, also acting as effectors. This is particularly well described in the case of VgrG1 and VgrG3 from Vibrio cholerae (21, 27, 55). VgrG1 has a C-terminal extension, described as an actin-cross-linking domain (31), which is used to impair the phagocytic activity of macrophages (27). More recently, VgrG3 was shown to carry a C-terminal extension which may act as a bacterial toxin using its hydrolase activity against the peptidoglycan (55). These two VgrG proteins are called evolved proteins, and only the Vibrio cholerae VgrG2 protein is canonical, i.e., it is restricted to the structure of the puncturing device of the bacteriophage (18), with no catalytic domain extension at the C terminus. In P. aeruginosa PAO1, there are 10 VgrG proteins, of which only VgrG2b could be predicted to have a C-terminal extension (19, 21). In PA14, the 11th VgrG protein, VgrG14, has no clear C-terminal extension, suggesting that it might not be an H2-T6SS effector *per se*. However, canonical VgrG proteins are also recovered in the culture supernatants of bacteria with active T6SS (19, 29, 59). We introduced a V5 tag at the VgrG14 C terminus to follow its fate by Western blotting, but we could not detect it in the supernatant. The lack of observed secretion can be discussed in various ways. The amount of VgrG14 protein detected in the cells was not very high, and previous studies have shown that the percentage of secreted VgrG proteins is rather low (19, 29). VgrG14 secretion could be undetected in this case. The addition of a C-terminal tag may have interfered with the puncturing device function, and therefore with H2-T6SS function. However, the observed Hcp2 secretion does not favor this hypothesis. Finally, additional genes outside the two arabinose-controlled clusters are required for VgrG secretion and were likely not expressed under our growth conditions.

Within the *vgrG* islands (36), additional genes could be found and happened to encode T6SS substrates. The observation that the gene downstream of P. aeruginosa *vgrG4b*, now called *tle5*, encodes a T6SS-dependent phospholipase with antibacterial activity is one such noteworthy example. The gene immediately downstream of *vgrG14* does not display any recognizable features. However, the one next to it encodes a protein of the Rhs family (41, 52), whose role and function have yet to be understood. Interestingly, a recent study identified one such protein, called RhsT, in the P. aeruginosa isolate PSE9 (56). RhsT can be translocated into J774 macrophage cells and kills them by influencing inflammasome signaling. However, it is not known whether RhsT is a substrate of the T6SS or any other known secretion systems. Another recent report described XadM from the plant pathogen *Xanthomonas oryzae* as a cell surface protein, a member of the Rhs family, and required for attachment to host cells, biofilm formation, and global virulence (60). We thus added a V5 tag at the RhsP2 C terminus and followed its secretion fate by Western blotting. Secretion could not be detected, but as discussed for VgrG14, several reasons may have prevented the detection of secreted RhsP2. It is interesting that in P. aeruginosa strain 39016, the H2-T6SS cluster does not include any *vgrG* or *hcp* genes but is associated with the *rhs* gene. If one considers that VgrG and Hcp are core components of the T6SS machine and that Rhs is a putative substrate, its secretion might require another *vgrG/hcp* subset localized elsewhere on the 39016 chromosome.

The P. aeruginosa PAO1 H2-T6SS has been shown to modulate bacterial internalization into nonphagocytic eukaryotic cells (50). Because PA14 is highly cytotoxic toward eukaryotic cells (40, 49), we engineered a T3SS mutant to study the impact of the PA14 H2-T6SS on internalization. Surprisingly, we observed the opposite effect compared to that with PAO1, and we concluded that in PA14 the H2-T6SS does not promote but prevents internalization. Although it is not straightforward to reconcile these data, it is clear that the function of a secretion system is given by the nature of its substrate/effector. For example, the pathogenesis mechanisms of PAO1 and PA14 are entirely different, since one strain, PAO1, is considered invasive and the other, PA14, is cytotoxic. Both have a T3SS, but the presence of an additional effector, ExoU (61), encoded on a pathogenicity island of PA14 (40), contributes to a change in lifestyle.

Previous observations suggesting that the H2-T6SS affects virulence (51), internalization (50), and bacterial killing (37) are somehow puzzling and reflect the pleiotropic role of this system, and likely a broad set of associated effectors. The discovery that the phospholipase Tle5, an effector linked with the H2-T6SS (37), is involved in bacterial killing gives further support to this hypothesis. Whereas it has still to be determined whether RhsP2 is an H2-T6SS substrate, the genetic difference between the H2-T6SS gene clusters of PAO1 and PA14 and the association of an *rhs*-like gene might account for a distinct phenotypic contribution to pathogenesis. Further analysis of the role and function of RhsP2 and the identification of the whole set of H2-T6SS-dependent effectors by secretome analysis are needed to obtain a comprehensive vision of H2-T6SS function and to shed more light on the multiple and central roles of T6SSs in bacterial pathogenesis.

## Supplementary Material

Supplemental material
