# Supplementary material for: An rhs Gene Linked to the Second Type VI Secretion Cluster Is a Feature of the Pseudomonas aeruginosa Strain PA14
Source: J Bacteriol. 2014 Feb;196(4):800–10. doi: 10.1128/JB.00863-13 (PMC3911176; doi:10.1128/JB.00863-13)
Supplement: Supplemental material [file supp_196_4_800__index.html]

An rhs Gene Linked to the Second Type VI Secretion Cluster Is a Feature of the Pseudomonas aeruginosa Strain PA14 — Supplemental material 

# An *rhs* Gene Linked to the Second Type VI Secretion Cluster Is a Feature of the Pseudomonas aeruginosa Strain PA14

## Supplemental material

**Files in this Data Supplement:**

- Supplemental file 1 -

  Fig. S1, secretion profile and cytotoxicity of a PA14 *pscC* mutant

  PDF, 414K
