# Supplementary material for: An rhs Gene Linked to the Second Type VI Secretion Cluster Is a Feature of the Pseudomonas aeruginosa Strain PA14
Source: J Bacteriol. 2014 Feb;196(4):800–10. doi: 10.1128/JB.00863-13 (PMC3911176; doi:10.1128/JB.00863-13)
Supplement: Supplemental material [file JB.00863-13_zjb999093027so1.pdf]

A

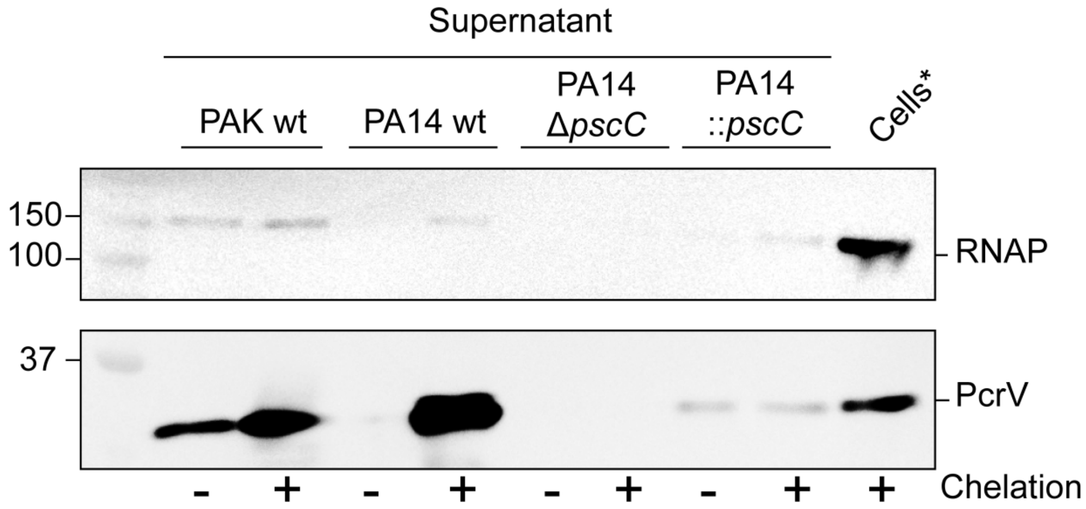

B

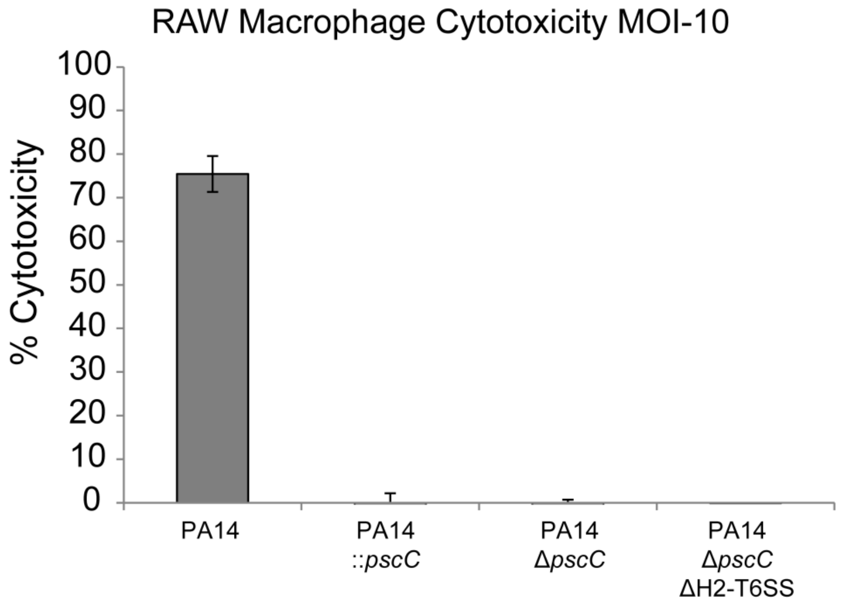

FIG S1 Secretion profile and cytotoxicity of a *pscC* mutant in PA14. (A) T3SS protein secretion profile in supernatants of wild type PAK, wild type PA14, a clean *pscC* deletion mutant, and a PA14::*pscC* transposon mutant, as indicated above the blots. Blots are probed with anti-RNAP (upper) or anti-PcrV (lower), with the expected position of each protein indicated on the right and molecular weight markers the left. The presence (+) or absence (-) of calcium chelation used to induce the T3SS is indicated below the blots. A calcium chelated whole cell extract of PAK is included as a positive control for the RNAP protein (*). (B) Cytotoxicity (LDH release assay) from RAW macrophages infected with strains indicated below the chart at an MOI of 10 during a 3 hour infection. Percentage of cytotoxicity is shown on the vertical axis. Error bars show standard deviation.
